# Structural Alterations in the Corpus Callosum Are Associated with Suicidal Behavior in Women with Borderline Personality Disorder

**DOI:** 10.3389/fnhum.2017.00196

**Published:** 2017-04-24

**Authors:** Alexander Lischke, Martin Domin, Harald J. Freyberger, Hans J. Grabe, Renate Mentel, Dorothee Bernheim, Martin Lotze

**Affiliations:** ^1^Department of Psychiatry and Psychotherapy, University of GreifswaldGreifswald, Germany; ^2^Department of Psychology, University of GreifswaldGreifswald, Germany; ^3^Functional Imaging Unit, Center for Diagnostic Radiology and Neuroradiology, University of GreifswaldGreifswald, Germany; ^4^Helios HospitalStralsund, Germany; ^5^Department of Child and Adolescent Psychiatry and Psychotherapy, University of UlmUlm, Germany

**Keywords:** borderline personality disorder, suicidal behavior, self-injurious behavior, emotion regulation, impulse control, corpus callosum, diffusion tensor imaging

## Abstract

Structural alterations in the corpus callosum (CC), the major white matter tract connecting functionally related brain regions in the two hemispheres, have been shown to be associated with emotional instability, impulsivity and suicidality in various mental disorders. To explore whether structural alterations of the CC would be similarly associated with emotional instability, impulsivity and suicidality in borderline personality disorder (BPD), we used diffusion tensor imaging (DTI) to assess the structural integrity of the CC in 21 BPD and 20 healthy control (HC) participants. Our hypothesis-driven analyses revealed a positive correlation between BPD participants’ suicidal behavior and fractional anisotropy (FA) in the splenium and genu of the CC and a negative correlation between BPD participants’ suicidal behavior and mean diffusivity (MD) in the splenium of CC. Our exploratory analyses suggested that suicidal BPD participants showed less FA and more MD in these regions than HC participants but that non-suicidal BPD participants showed similar FA and MD in these regions as HC participants. Taken together, our findings suggest an association between BPD participants’ suicidal behavior and structural alterations in regions of the CC that are connected with brain regions implicated in emotion regulation and impulse control. Structural alterations of the CC may, thus, account for deficits in emotion regulation and impulse control that lead to suicidal behavior in BPD. However, these findings should be considered as preliminary until replicated and extended in future studies that comprise larger samples of suicidal and non-suicidal BPD participants.

## Introduction

The corpus callosum (CC) is the largest white matter tract in the human brain, connecting functionally related brain regions in the two hemispheres (Gazzaniga, 2000). The CC contains between 200 and 800 million fibers that are coarsely organized in a topographical fashion (Aboitiz et al., 1992a,b). Small-diameter fibers, which are more common in the genu and splenium of the CC, connect prefrontal and tempo-parietal brain regions, whereas large diameter fibers, which are more common in the body and isthmus of the CC, connect visual, auditory and somatosensory regions. The CC is, thus, crucially implicated in the integration of inter-hemispheric information, which is essential for the effective coordination of cognition, emotion and behavior (Gazzaniga, 2000). Structural alterations of the CC may lead to impairments in inter-hemispheric communication, which may account for a variety of cognitive, emotional and behavioral disturbances (Gazzaniga, 2000). Impaired inter-hemispheric communication has, in fact, been found to be related to cognitive, emotional and behavioral disturbances, including severe ones, like, for example, suicidal behavior (Amen et al., 2009; Matsuo et al., 2010; Cyprien et al., 2011). It is, therefore, not surprising that structural alterations of the CC are quite prevalent among mental disorders that are characterized by marked deficits in emotion regulation and impulse control, such as bipolar disorder (BD; Brambilla et al., 2003; Emsell et al., 2013) or attention-deficit/hyperactivity disorder (ADHD; Luders et al., 2009; Dramsdahl et al., 2012). Borderline personality disorder (BPD), which shares common features with BD (Bassett, 2012) and ADHD (Philipsen, 2006), is also characterized by deficits in emotion regulation and impulse control (Henry et al., 2001; Krause-Utz et al., 2013). Moreover, deficits in emotion regulation and impulse control may even lead to self-injurious and suicidal behavior in BPD (Yen et al., 2004; Kleindienst et al., 2008; Wedig et al., 2012; Boisseau et al., 2013). Structural alterations in the CC may, thus, also be related to emotional instability and impulsivity in BPD. However, structural alterations of the CC have rarely been studied in BPD (Rüsch et al., 2007a; Zanetti et al., 2007; García-Bueno et al., 2008; Gan et al., 2016), leaving open whether this is indeed the case. An early study found no differences in the size and shape of the CC between BPD participants and healthy control (HC) participants (Zanetti et al., 2007), challenging the notion of structural alterations of the CC in BPD. A latter study, on the contrary, revealed a reduction of callosal thickness in BPD as compared to HC participants (Rüsch et al., 2007a), lending support for this notion. Of note, cortical thickness analyses are more sensitive to microscopic structural alterations than shape or size analyses (Hofer and Frahm, 2006), suggesting that micro- rather than macroscopic alterations of the CC are implicated in BPD participants’ deficits in emotion regulation and impulse control. Recent studies investigating microscopic alterations of CC, in fact, found structural differences between BPD and HC participants (Carrasco et al., 2012; Gan et al., 2016).

Motivated by these findings (Carrasco et al., 2012; Gan et al., 2016), we used diffusion tensor imaging (DTI) to further investigate microscopic alterations of the CC in BPD. DTI allows the measurement of the magnitude and direction of water diffusion in white matter tracts like the CC, thereby providing information about white matter integrity (Mori and Zhang, 2006). We used two common measures, fractional anisotropy (FA), and mean diffusivity (MD), to characterize the myelination, orientation and density of fibers in the CC. FA represents the coherence of water diffusion in fibers, whereas MD represents the overall water diffusion in fibers (Mori and Zhang, 2006). Microscopic alterations of the CC are, thus, indicated by low FA and high MD in the corresponding fibers. BPD participants may show lower FA and higher MD in the CC than HC participants (Carrasco et al., 2012; Gan et al., 2016). Moreover, these difference may be more pronounced between suicidal BPD participants and HC participants than between non-suicidal BPD participants and HC participants (Amen et al., 2009; Matsuo et al., 2010; Cyprien et al., 2011). Taking the topographical organization of the CC into account (Aboitiz et al., 1992a,b), differences in FA and MD may be most pronounced in regions of the CC that are connected with brain regions implicated in emotion regulation and impulse control (Westerhausen et al., 2003; Matsuo et al., 2010; Cyprien et al., 2011; Carrasco et al., 2012; Gan et al., 2016). We, thus, expected FA and MD in the splenium and genu, but not in the body, of the CC to be associated with measures that indicate deficits in emotion regulation and impulse control, in particular with measures indicating suicidal behavior (Amen et al., 2009; Matsuo et al., 2010; Cyprien et al., 2011). Being aware of the fact that BPD is a highly heterogeneous disorder (Paris, 2005), we tested these predictions in a well-defined sample of HC and BPD participants following procedures employed in previous studies investigating structural alterations in BPD (Rüsch et al., 2007a,b, 2010).

## Materials and Methods

### Participants

Twenty healthy women (HC) and 21 women with a DSM-IV diagnosis of BPD participated in the study (see Table 1). BPD participants were included in the study if they met DSM-IV criteria for a diagnosis of BPD, including criteria associated with emotional instability and impulsivity. In addition, BPD participants had to meet DSM-IV criteria for ADHD, including criteria associated with impulsivity. BPD participants who met DSM-IV diagnostic criteria for substance dependance, BD, schizoaffective disorder, schizophrenia or schizotypical personality disorder were excluded from the study. BPD participants taking typical anti-psychotic medication were also excluded. Included BPD participants, thus, formed a representative and homogeneous sample of BPD participants that was characterized by marked deficits in emotion regulation and impulse control. Accordingly, all BPD participants reported acts of self-injurious behavior and most BPD participants reported acts of suicidal behavior (see Supplemental Material S1, Supplementary Tables S1, S2). Exclusion criteria for HC participants were a diagnosis of any mental disorder according to DSM-IV criteria. HC participants were also excluded if they met DMS-IV criteria for emotional instability and impulsivity as described in the DSM-IV guidelines for BPD and ADHD. Additional exclusion criteria for all participants were related to factors that are known to affect the structural integrity of the CC, such as age (Lebel et al., 2010), sex (Menzler et al., 2011) and handedness (Westerhausen et al., 2003). Consequently, all participants were right-handed women with an age range of 18–45 years. Besides this, BPD and HC participants were matched for age, education and intelligence to control for structural differences in the CC that were not related to deficits in emotional regulation and impulse control.

**Table 1:** **Differences in demographical and psychopathological measures between HC and BPD participants**.

	HC (*n* = 20)		BPD (*n* = 21)		Test statistic	
	*n*	*%*	*n*	*%*	$\chi_{\left(2,\;N=41\right)}^{2}$	*p*
Education					2.00	0.367
Basic	0	0.00	1	0.05
Intermediate	5	0.25	8	0.38
Advanced	15	0.75	12	0.57
	*M*	*SD*	*M*	*SD*	*F*_(1,39)_	*p*
Age	26.81	4.89	26.21	6.12	0.12	0.731
Intelligence (MWT-B-IQ)	108.20	9.20	108.76	14.60	0.02	0.884
Borderline personality (BSL-23)	1.55	2.09	40.76	20.40	73.07***	<0.001
Attention deficit/Hyperactivity (ADHD-SR)	1.95	1.70	22.24	9.29	92.24***	<0.001
Impulsivity (BIS-11)	52.85	7.69	74.43	12.09	45.96***	<0.001
Anger (STAXI-T)	13.50	2.48	26.43	7.49	53.93***	<0.001
Anxiety (STAI-T)	29.25	4.81	61.43	12.62	114.06***	<0.001
Depression (BDI)	2.20	2.93	30.76	14.29	76.77***	<0.001

Note: HC, healthy control participants; BPD, borderline personality disorder participants; MWT-IQ, Multiple choice vocabulary test—Intelligence quotient (Lehrl et al., 1995); BSL-23, Borderline Symptom List 23 (Bohus et al., 2009); ADHD-SR, Attention Deficit Hyperactivity Disorder—Self Report Scale (Rösler et al., 2004); BIS-11, Barratt Impulsiveness Scale Version 11 (Preuss et al., 2008); STAXI-T, State Trait Anger Expression Inventory—Trait Version (Schwenkmezger et al., 1992); STAI-T, State Trait Anxiety Inventory—Trait Version (Laux et al., 1981); BDI, Beck Depression Inventory (Hautzinger et al., 1995). ***p < 0.001.

All participants provided written informed consent and were reimbursed for their time. This study was carried out in accordance with the recommendations of ethics comitee of the University of Greifswald with written informed consent from all subjects. All subjects gave written informed consent in accordance with the Declaration of Helsinki. The protocol was approved by the ethics committee of the University of Greifswald.

### Measures

Clinical diagnoses of BPD and ADHD were validated with structured clinical interviews (Structured Clinical Interview for DSM-IV Axis II Disorders, SCID-II; Fydrich et al., 1997; Composite International Diagnostic Interview, CIDI; Wittchen and Pflister, 1997) and questionnaires (Borderline Symptom List, BSL; Bohus et al., 2009; ADHD-Self Report Scale (SR); Rösler et al., 2004). Mental disorders other than BPD or ADHD were also assessed with structured clinical interviews (CIDI; Wittchen and Pflister, 1997). Clinical assessment of (para-)suicidal behavior was validated with an in-house questionnaire measuring self-injurious behavior with and without suicide intent or expectation of death (see Supplemental Material S1; Self-harm Suicide and Medication Questionnaire, SVUM; Barnow et al., 2014). Further questionnaires were used to assess deficits in emotion regulation and impulse control (BSL; Bohus et al., 2009; Barratt Impulsiveness Scale Version 11, BIS-11; Preuss et al., 2008; ADHD-SR; Rösler et al., 2004). Feelings of depression (Beck Depression Inventory, BDI; Hautzinger et al., 1995), anxiety (State-Trait Anxiety Inventory, STAI-T, Laux et al., 1981) and anger (State-Trait Anger Expression Inventory, STAXI-T; Schwenkmezger et al., 1992) were also assessed with questionnaires. Intelligence was assessed with a multiple choice vocabulary test (Multiple Choice Vocabulary Test, MWT; Lehrl et al., 1995).

### Magnetic Resonance Imaging

#### Data Acquisition

Magnetic resonance imaging was performed on a 3-Tesla whole-body MR-scanner (Verio, Siemens, Erlangen, Germany) equipped with a 32-channel head coil. Diffusion-weighted images were recorded with a multi directional diffusion weighting (MDDW) sequence (repetition time (TR): 15300 ms; echo time (TE): 107 ms; flip angle (FA): 90°; field of view (FOV): 230 × 230 mm; Matrix: 128 × 128). In all, 80 axial slices (slice thickness: 2 mm, no gap; voxel size: 1.8 × 1.8 × 2 mm^3^) were acquired along 64 gradient directions with two b values (0 and 1000 mm/s^2^) and one repetition. Additionally, high-resolution structural images were recorded with a T1-weighted coronal oriented magnetization-prepared rapid gradient echo (MPRAGE) sequence (TR: 2900 ms; TE: 2.52 ms; FA: 25°; FOV, 256 × 256 mm; Matrix: 256 × 256), leading to the acquisition of 176 sagittal slices (voxel size: 1 × 1 × 1 mm^3^).

#### Data Processing

**Figure 1:**
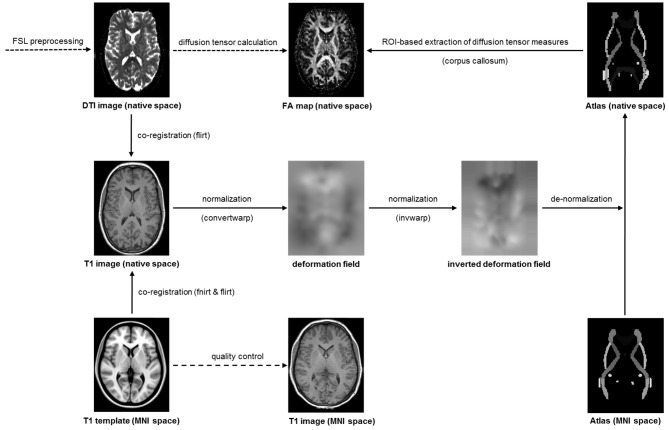
**Processing and analysis stream for the diffusion weighted images**.

Following established procedures (Domin et al., 2014), the diffusion-weighted images were preprocessed with tools from the Functional Magnetic Resonance Imaging of the Brain Software Library^1^ (see Figure 1). After correction of eddy currents and head motion, skull stripping was performed with *bet*. Next, *flirt* was used for a linear co-registration of the diffusion-weight image and the structural image as well as for a linear co-registration of the structural image and the MNI template. Then, *fnirt* was used for a non-linear co-registration of the structural image and the MNI template. The transformation information obtained during the linear and non-linear co-registration was combined using *convertwarp* and subsequently inversed using *invwarp*. The inversed transformation information was used for the de-normalization of the ICBM-DTI-81 white-matter labels atlas (Mori et al., 2008; Oishi et al., 2008), which contained anatomical masks in MNI space. De-normalization of this atlas was necessary because the diffusion-weighted images were deliberately left in native space to avoid unnecessary image transformations during normalization to MNI space. Finally, the gradient vectors for the diffusion weighting gradients were corrected by the rotation matrix calculated during motion correction and linear co-registration.

#### Data Analysis

JavaDTI, an in-house software package (Domin et al., 2014), was used for diffusion tensor calculation and deterministic diffusion tensor tractography. For diffusion tensor calculation, a diffusion tensor model was fitted at each voxel to provide a voxelwise calculation of FA and MD (Le Bihan et al., 2001). For deterministic diffusion tensor tractography, tracts were determined by a continuous path originating from a seed voxel in a region of interest and following the tensor direction (Mori et al., 1999). Parameter thresholds for tract determination were FA values greater than 0.20 and turning angles smaller than 45°. Tracts were determined in pre-defined regions of the CC using de-normalized masks of the ICBM-DTI-81 white-matter labels atlas (Mori et al., 2008; Oishi et al., 2008). That is, the genu, splenium and body of the CC. For each of these tracts, the mean FA as well as the mean MD were computed and extracted for the statistical analyses.

#### Data Visualization

**Figure 2:**
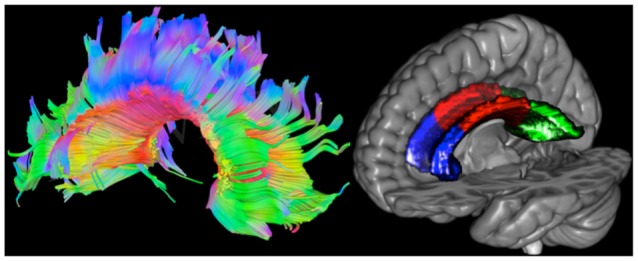
**Left: tracts of the corpus callosum (CC) projected on the diffusion weighted image of one healthy control (HC) participant**. Red, green and blue represent left to right, anterior to posterior and superior to inferior directions, respectively. **Right**: masks of the CC projected on the Collins MNI-reference brain. The genu is colored in blue, the body in red and the splenius in green.

For visualization purposes (see Figure 2), tracts of the CC were projected on the diffusion weighted image of one HC participant using DSI Studio^2^.

### Statistical Analyses

All statistical analyses were conducted using SPSS 22 (SPSS Inc., Chicago, IL, USA). To describe participants with respect to their demographical and psychopathological characteristics, differences in demographical and psychopathological measures between HC and BPD participants were analyzed with chi-square tests and one-way analysis of variances (ANOVAs). To investigate associations between microscopic alterations of the CC and psychopathological characteristics, zero-order correlations between diffusion tensor measures and psychopathological measures were analyzed, separately for HC and BPD participants. Two sets of analyses were conducted: the first set of analyses tested *a priori* predictions regarding the association between suicidal behavior and structural alterations in distinct regions of the CC, namely the splenium and genu as compared to the body of the CC (Amen et al., 2009; Matsuo et al., 2010; Cyprien et al., 2011). The second set of analyses explored whether deficits in emotion regulation and impulse control, which are precursors of suicidal behavior in BPD (Yen et al., 2004; Boisseau et al., 2013), would be similarly associated with structural alterations in these callosal regions. Besides this, one-way ANOVAs and planned comparisons were used to explore whether the predicted associations between suicidal behavior and structural alterations would be reflected in structural differences between HC participants and BPD participants with or without suicidal behavior. The significance level for all analyses was set at *p* < 0.05 (two-tailed). In addition to the significance level *p*, effect sizes (*d* and *r*) are reported to facilitate the interpretation of significant results.

## Results

### Differences in Demographical and Psychopathological Measures

To investigate whether HC and BPD participants differed in their demographical and psychopathological measures, chi-square tests and one-way ANOVAs were run (see Table 1). There were no differences in age, education or intelligence between HC and BPD participants. However, BPD participants reported more deficits in emotion regulation and impulse control on the BSL-23, ADHD-SR and BIS-11 than HC participants. BPD participants also reported more feelings of depression, anxiety and anger on the BDI, STAI-T and STAXI-T than HC participants. Of note, differentiating between suicidal and non-suicidal BPD participants in these analyses revealed a similar pattern of results (see Supplemental Material S2, Supplementary Table S3).

### Associations between Diffusion Measures and Psychopathological Measures

**Figure 3:**
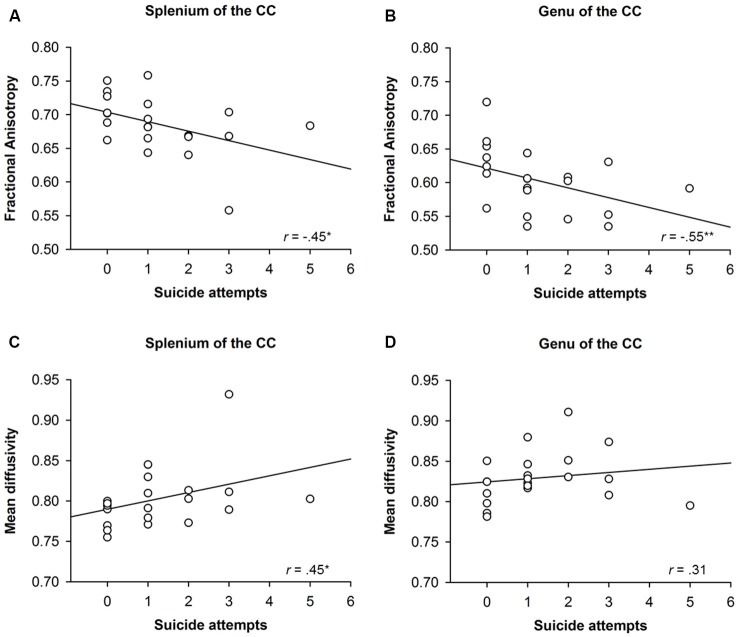
**Correlation between structural alterations in distinct regions of the Corpus Callosum (CC) and suicidal behavior of participants with borderline personality disorder (BPD)**. The upper panels **(A,B)** show the correlation between suicidal attempts and fractional anisotropy (FA) in the splenium and genu of the CC, whereas the lower panels **(C,D)** show the correlation between suicide attempts and mean diffusivity (MD) in the splenium and genu of the CC. *p < 0.05, **p < 0.01.

To investigate whether structural alterations of the CC were associated with BPD participants’ suicidal behavior, zero-order correlations were run between FA and MD in distinct regions of the CC and measures of suicidal behavior like the SVUM (see Figure 3). The number of suicide attempts correlated with FA (*r* = −0.45, *p* = 0.04) and MD (*r* = 0.45, *p* = 0.04) in the splenium as well as with FA (*r* = −0.55, *p* = 0.01), but not MD (*r* = 0.31, *p* = 0.18), in the genu of the CC. FA (*r* = −0.25, *p* = 0.28) and MD (*r* = 0.28, *p* = 0.22) in the body of the CC, on the contrary, were not correlated with the number of suicide attempts.

To explore possible associations between structural alterations in the CC and deficits in emotion regulation and impulse control, zero-order correlations were run between FA and MD in distinct regions of the CC and measures indicating emotional instability and impulsivity, such as the BSL-23, ADHD-SR and BIS-11. Deficits in emotion regulation and impulse control were uncorrelated with FA or MD in any region of the CC, among BPD participants (all *r* < |0.30|, all *p* > 0.20) as well as among HC participants (all *r* < |0.20|, all *p* > 0.39).

### Differences in Diffusion Measures

To explore whether HC participants and BPD participants with or without suicidal behavior showed differences in FA and MD in distinct callosal regions, one-way ANOVAs were run (see Table 2). Across participants, there were no differences in FA and MD in the body and genu but in the splenium of the CC. To elucidate whether the differences in FA and MD in the splenium of the CC were due to differences between BPD participants with suicidal behavior and HC participants or due to differences between BPD participants without suicidal behavior and HC participants, planned comparisons were run. Suicidal BPD participants showed lower FA (*p* = 0.02, *d* = 0.86) and, at least on a trend level, higher MD (*p* = 0.05, *d* = −0.68) in the splenium of the than HC participants. Non-suicidal BPD participants, on the contrary, showed comparable FA (*p* = 0.90) and MD (*p* = 0.18) in the splenium of the CC as HC participants. However, the aforementioned differences in FA and MD between BPD participants with suicidal behavior and HC participants should be interpreted with caution because only a small number of participants were investigated in the respective analysis.

**Table 2:** **Differences in diffusion measures between HC and BPD participants with or without suicidal behavior**.

	HC (*n* = 20)		BPD-SA (*n* = 8)		BPD+SA (*n* = 13)		Test statistic	
	*M*	*SD*	*M*	*SD*	*M*	*SD*	*F*_(1,39)_	*p*
FA in distinct regions of the CC
Genu	0.60	0.05	0.63	0.05	0.58	0.04	2.91	0.07
Splenium	0.70	0.03	0.71	0.03	0.67	0.05	3.51*	0.04
Body	0.61	0.04	0.62	0.04	0.59	0.05	1.39	0.26
MD in distinct regions of the CC
Genu	0.82	0.03	0.82	0.03	0.84	0.03	1.53	0.23
Splenium	0.79	0.03	0.78	0.02	0.82	0.04	3.86*	0.03
Body	0.80	0.04	0.80	0.03	0.82	0.04	2.37	0.11

Note: HC, healthy control participants; BPD-SA, borderline personality disorder participants without suicidal behavior; BPD+SA, borderline personality disorder participants with suicidal behavior; FA, fractional anisotropy; MD, mean diffusivity; CC, corpus callosum. *p < 0.05.

## Discussion

In the present study, we used DTI to investigate the structural integrity of the CC, the major white matter tract connecting functionally related brain regions in the two hemispheres (Gazzaniga, 2000), in BPD. Previous studies revealed associations between structural alterations in the CC and deficits in emotion regulation and impulse control (Brambilla et al., 2003; Luders et al., 2009; Carrasco et al., 2012; Dramsdahl et al., 2012; Emsell et al., 2013; Gan et al., 2016), including severe ones like, for example, suicidal behavior (Amen et al., 2009; Matsuo et al., 2010; Cyprien et al., 2011). As BPD is characterized by marked deficits in emotion regulation and impulse control, which often lead to suicidal behavior (Yen et al., 2004; Boisseau et al., 2013), we expected to find similar associations in BPD but not in HC participants. More precisely, measures of emotional instability and impulsivity, in particular those indicating suicidal behavior, were expected to be associated with FA and MD in the splenium and genu of the CC. Of note, these measures were not expected to be associated with FA and MD in the body of the CC because only the genu and splenium of the CC are connected with brain regions implicated in emotion regulation and impulse control (Aboitiz et al., 1992a,b). Besides this, we expected FA and MD in these callosal regions to differ between HC participants and BPD participants with suicidal behavior but not between HC participants and BPD participants without suicidal behavior.

As predicted, we found associations between structural alterations in the CC and measures of suicidal behavior in BPD participants. FA in the splenium of the CC correlated negatively with suicidal behavior, whereas MD in the splenium of the CC correlated positively with suicidal behavior. Moreover, FA in the genu of the CC also correlated negatively with suicidal behavior. FA or MD in the body of the CC, on the contrary, was uncorrelated with suicidal behavior. Our findings regarding structural alterations in the CC of BPD participants with and without suicidal behavior were also consistent with our predictions. Suicidal BPD participants showed lower FA and higher MD in the splenium of the CC than HC participants, whereas non-suicidal BPD participants showed comparable FA and MD in the splenium of the CC as HC participants. However, these structural differences should be interpreted with caution because only a small number of BPD participants with and without suicidal behavior were investigated in the respective analyses. In this regard, it is noteworthy that recent studies investigating larger number of BPD participants also revealed structural alterations in regions of the CC that are connected with brain regions implicated in emotion regulation and impulse control (Carrasco et al., 2012; Gan et al., 2016). Structural alterations in the splenium of the CC, as suggested by the present findings, and structural alterations in the genu of the CC, as suggested by previous findings (Carrasco et al., 2012; Gan et al., 2016), may, thus, account for emotional instability, impulsivity and suicidality in BPD.

Although we had *a priori* predictions regarding the association between structural alterations in the CC and measures of suicidal behavior in BPD participants, we also explored whether measures indicating deficits in emotion regulation and impulse control, would be similarly associated with structural alterations in the CC. However, none of these measures correlated substantially with FA or MD in the CC. It may be possible that we would have obtained more substantial correlations between structural alterations and emotional instability and impulsivity, if we had employed measures that assess deficits in emotion regulation and impulse control that are more closely related to suicidal behavior (Yen et al., 2004; Boisseau et al., 2013).

Overall, our findings indicate that structural alterations in the splenium and genu of the CC, which are connected with brain regions implicated in emotion regulation and impulse control (Aboitiz et al., 1992a,b), are associated with suicidal behavior in BPD. It is interesting to note that structural alterations in these regions have also been found in other disorders than BPD, in particular in those that are characterized by deficits in emotion regulation and impulse control (Wang et al., 2008; Matsuo et al., 2010; Cyprien et al., 2011; Dramsdahl et al., 2012; Emsell et al., 2013). Moreover, these alterations have also been shown to be associated with suicidal behavior (Amen et al., 2009; Matsuo et al., 2010; Cyprien et al., 2011). Structural alterations in the genu and splenium of the CC, thus, appear to account for suicidal behavior in various disorders, presumably due to general rather than disorder-specific deficits in emotion regulation and impulse control (Jollant et al., 2011). However, patients engaging in suicidal behavior do not only show deficits in emotion regulation and impulse control but also deficits in problem-solving (Pollock and Williams, 1998). The latter deficits may often be a consequence of the former deficits, indicating that deficits in problem-solving may mediate the effects of emotional instability and impulsivity on suicidal behavior. In this regard it is noteworthy that the genu and splenium of the CC contain fibers that connect prefrontal and tempo-parietal brain regions that are implicated in emotion regulation and impulse control (Ochsner and Gross, 2005) as well as prefrontal and tempo-parietal brain regions that are implicated in problem-solving (Jung and Haier, 2007). Structural alterations in the genu and splenium of the CC may, thus, not only account for deficits in emotion regulation and impulse control but also for deficits in problem-solving, thereby increasing the risk for suicidal behavior in a general rather than disorder-specific way (Jollant et al., 2011).

Our findings regarding structural alterations in different regions of the CC may help to further our understanding of the neurobiological underpinnings of BPD. The structural alterations were most pronounced in regions of the CC that are connected with prefrontal and tempo-parietal brain regions (Aboitiz et al., 1992a,b). Functional and structural alterations in these prefrontal and tempo-parietal brain regions, which are crucially implicated in emotion regulation and impulse control (Ochsner and Gross, 2005), have repeatedly been demonstrated in BPD (Krause-Utz et al., 2014). It may, thus, be possible that structural alterations in the splenium and genu of the CC lead to functional and structural alterations in prefrontal and tempo-parietal brain regions, such as the amygdala (Herpertz et al., 2001; Schmahl et al., 2003; Tebartz van Elst et al., 2003; Koenigsberg et al., 2009a; Schulze et al., 2011; Niedtfeld et al., 2013), hippocampus (Schmahl et al., 2003; Tebartz van Elst et al., 2003; Radaelli et al., 2012; Niedtfeld et al., 2013), anterior cingulate cortex (Tebartz van Elst et al., 2003; Hazlett et al., 2005; Koenigsberg et al., 2009a; Rüsch et al., 2010; Niedtfeld et al., 2013), dorsolateral and ventromedial prefrontal cortex (Tebartz van Elst et al., 2003; Rüsch et al., 2007b; Koenigsberg et al., 2009b; Schulze et al., 2011; Bruehl et al., 2013). Future studies should, therefore, combine functional and structural techniques to further elucidate the neurobiological underpinnings of BPD.

Compared to similar studies investigating structural alterations in BPD, our study has some strengths and weaknesses. In contrast to previous studies (Carrasco et al., 2012; Gan et al., 2016), we investigated a highly homogenous and representative sample of BPD participants. Previous studies investigated male and female BPD participants with less pronounced deficits in emotion regulation and impulse control because participants with chronic conditions or acute conditions that could not be stabilized throughout treatment were not considered for recruitment (Carrasco et al., 2012; Gan et al., 2016). We, on the contrary, recruited female BPD participants with a long-standing history of emotional instability and impulsivity that were not stabilized at the time of the study. Mirroring BPD participants’ deficits in emotion regulation and impulse control, self-injurious and suicidal behavior was quite prevalent among BPD participants. Although the prevalence of self-injurious and suicidal behavior was comparable to those typically found in BPD samples (Yen et al., 2004; Kleindienst et al., 2008; Wedig et al., 2012; Boisseau et al., 2013), the number of reported suicide attempts was rather low. Consequently, all findings regarding BPD participants’ suicidal behavior should be regarded as preliminary until replicated and extended in future studies. It should be noted, however, that the association between suicidal behavior and structural alterations in distinct regions of the CC was evident across a series of analyses. Although these analyses yielded large effect sizes (effect size for differences in structural integrity of HC and BPD participants ranging from* d* = |0.68| to *d* = |0.86|; effect size for associations between structural integrity and suicide attempts among BPD participants ranging from *r* = |0.45| to *r* = |0.55|), it remains to be determined whether future studies will report effect sizes of similar magnitude. Future studies may benefit from utilizing a similar approach to data analysis as the one employed in present study. In contrast to previous studies (Carrasco et al., 2012; Gan et al., 2016), we followed a strictly hypothesis-driven approach in our analysis. Our focus was on brain regions that have previously been shown to be associated with emotional instability, impulsivity and suicidality. Consequently, we tested *a priori* predictions regarding associations between structural alterations in pre-defined regions of interest and measures indicating deficits in emotion regulation and impulse control. Also in contrast to previous studies (Carrasco et al., 2012; Gan et al., 2016), we employed novel imaging techniques to enhance the accuracy of our data analysis (e.g., acquiring diffusion weight images with small voxel size to increase the spatial resolution of possible alterations, analyzing diffusion weight images in native space to avoid unnecessary image transformations). Future studies should, therefore, combine standard and novel approaches to data analysis to further investigate the association between suicidal behavior and structural alterations in the CC, preferably in large sample of male and female participants suffering from various mental disorders to clarify the specificity of the present and previous findings (Rüsch et al., 2007a, 2010; Zanetti et al., 2007; Carrasco et al., 2012; Gan et al., 2016).

To summarize, we used DTI to investigate whether structural alterations of the CC would be associated with deficits in emotion regulation and impulse control, in particular with those that are related to suicidal behavior, in BPD. Among BPD participants, suicidal behavior was associated with structural alterations in regions of the CC that are connected with prefrontal and tempo-parietal brain regions implicated in emotion regulation, impulse control and problem-solving. Compared to HC participants, BPD participants with suicidal behavior showed more structural alterations in these regions than BPD participants without suicidal behavior. Structural alterations in distinct regions of the CC may, thus, account for deficits in emotion regulation and impulse control that ultimately lead to suicidal behavior in BPD.

## Author Contributions

AL, DB, HJF and ML designed the study. DB, HJG and RM recruited the participants and collected the data. AL and MD analyzed the data. AL wrote the manuscript. DB, MD, ML, HJF, HJG and RM contributed to writing, reviewing and editing of the manuscript. All authors approved the final version of the manuscript.

## Supplementary Material

The Supplementary Material for this article can be found online at: http://journal.frontiersin.org/article/10.3389/fnhum.2017.00196/full#supplementary-material

## Conflict of Interest Statement

The authors declare that the research was conducted in the absence of any commercial or financial relationships that could be construed as a potential conflict of interest.

## Footnotes

^1^http://www.fmrib.ox.ac.uk/fsl

^2^http://dsi-studio.labsolver.org/
