# Supplementary material for: Structural Alterations in the Corpus Callosum Are Associated with Suicidal Behavior in Women with Borderline Personality Disorder
Source: Front Hum Neurosci. 2017 Apr 24;11:196. doi: 10.3389/fnhum.2017.00196 (PMC5401902; doi:10.3389/fnhum.2017.00196)
Supplement: Supplementary file 1 [file Table_1.doc]

***Supplementary Material***

**Structural alterations in the corpus callosum are associated with suicidal behavior in women with borderline personality disorder**

**Alexander Lischke^1,2,3^, PhD; Martin Domin^3^, PhD; Harald J. Freyberger^1,4^, MD; Hans J. Grabe^1,4^, MD; Renate Mentel^1^, MD; Dorothee Bernheim^5*^, PhD; & Martin Lotze^3*^, MD**

^1^Department of Psychiatry and Psychotherapy, University of Greifswald, Greifswald, Germany

^2^Department of Psychology, University of Greifswald, Greifswald, Germany

^3^Functional Imaging Unit, Center for Diagnostic Radiology and Neuroradiology, University of Greifswald, Greifswald, Germany

^4^Helios Hospital, Stralsund, Germany

^5^Department of Child and Adolescent Psychiatry and Psychotherapy, University of Ulm, Ulm, Germany

*These authors contributed equally.

**Correspondence** concerning this article should be addressed to:

Alexander Lischke, Department of Psychology, University of Greifswald, Franz-Mehring-Str. 47, D-17489 Greifswald, Germany. Email: [alexander.lischke@uni-greifswald.de](mailto:alexander.lischke@uni-greifswald.de)

**S1 Prevalence of self-injurious and suicidal behavior among BPD participants**

Mirroring BPD participants’ emotional instability and impulsivity, self-injurious and suicidal behavior was quite prevalent among BPD participants (see Table S1 and Table S2). In fact, all BPD participants reported acts of self-injurious behavior and most BPD participants reported acts of suicidal behavior. Self-injurious behavior first occurred in the beginning of adolescence and comprised various self-injurious acts, with cutting and scratching being the most common ones (see Table S1). Suicidal behavior first occurred at the end of adolescence and involved various suicidal acts, with poisoning being the most common ones (see Table S2). Helplessness and desperation as well as pain and sorrow were the most common reasons for suicidal behavior (see Table S2).

**Table S1**

*Self-injurious behavior of BPD participants*

|  | | | *N* | | *%* |
| --- | --- | --- | --- | --- | --- |
| Number of participants committing self-injurious acts | | 21 | | 100.00 | |
| Number of participants not committing self-injurious acts | | 0 | | 0.00 | |
|  | | | *M* | | *SD* |
| Age at first self-injurious act | | | 13.24 | | 2.62 |
| Number of self-injurious acts | | | 21.52 | | 43.12 |
|  | Moderate acts | | 18.71 | | 35.17 |
|  | Severe acts | | 2.81 | | 10.86 |
|  | | | *N* | | *%* |
| Type of self-injurious acts | | |  | |  |
|  | Cutting | | 16 | | 76.20 |
|  | Scratching | | 16 | | 76.20 |
|  | Stabbing | | 2 | | 9.50 |
|  | Biting | | 5 | | 23.80 |
|  | Burning | | 3 | | 14.30 |
|  | Beating | | 8 | | 38.10 |
|  | Bone breaking | | 2 | | 9.50 |
|  | Hair pulling | | 3 | | 14.30 |
|  | Interfering with wound healing | | 8 | | 38.10 |
|  | Other | | 3 | | 14.30 |
| *Note.* Self-injurious behavior was assessed with an in-house questionnaire, the Self-harm, Suicide and Medication Questionnaire . The SVUM assesses the onset, frequency, type and severity of self-injurious acts that were committed without suicide intent or expectation of death. The focus of the SVUM is on self-injurious acts that were committed within the last six months. | | | | | |

**Table S2**

*Suicidal behavior of BPD participants*

|  | | *N* | *%* |
| --- | --- | --- | --- |
| Number of participants committing suicidal acts | | 13 | 61.90 |
| Number of participants not committing suicidal acts | | 8 | 38.10 |
|  | | *M* | *SD* |
| Age at first suicidal act | | 17.62 | 6.21 |
| Number of suicidal acts | | 1.29 | 1.35 |
|  | Moderate acts | 1.23 | 1.36 |
|  | Severe acts | 0.77 | 0.73 |
|  | | *N* | *%* |
| Type of suicidal acts | |  |  |
|  | Poisoning | 10 | 76.92 |
|  | Cutting | 1 | 7.69 |
|  | Jumping | 1 | 7.69 |
|  | Drowning | 1 | 7.69 |
|  | Strangling | 1 | 7.69 |
|  | Starving | 1 | 7.69 |
|  | Other | 2 | 15.38 |
|  | | *N* | *%* |
| Reasons for suicidal acts | |  |  |
|  | Helplessness and desperation | 13 | 100.00 |
|  | Pain and sorrow | 12 | 92.30 |
|  | Worthlessness and guilt | 4 | 30.77 |
|  | Anger and rage | 4 | 30.77 |
|  | Loneliness and isolation | 2 | 15.38 |
|  | Other | 1 | 7.69 |
| *Note.* Suicidal behavior was assessed with an in-house questionnaire, the Self-harm, Suicide and Medication Questionnaire . The SVUM assesses the onset, frequency, type and severity of and reasons for self-injurious acts that were committed with suicide intent or expectation of death. The focus of the SVUM is on suicidal acts that were committed across the life span. | | | |

**S2 Differences in demographical and psychopathological measures between HC participants and BPD participants with or without suicidal behavior.**

Chi-Square test and one-way ANOVAs with planned comparisons were run to investigate differences in demographical and psychopathological measures between HC participants and BPD participants with (BPD+SA) or without (BPD-SA) suicidal behavior (see Table S3). There were no differences in age, intelligence or education between HC participants and suicidal or non-suicidal BPD participants. Suicidal as well as non-suicidal BPD participants reported more deficits in emotion regulation and impulse control on the BSL-23 [BPD+SA vs. HC: *p* < .001, *d* = 4.34; BPD-SA vs. HC: *p* < .001, *d* = 5.24], ADHD-SR [BPD+SA vs. HC: *p* < .001, *d* = 4.76; BPD-SA vs. HC: *p* < .001, *d* = 6.18] and BSI-11 [BPD+SA vs. HC: *p* < .001, *d* = 2.41; BPD-SA vs. HC: *p* < .001, *d* = 2.24] than HC participants. Suicidal and non-suicidal BPD participants also reported more feelings of depression, anger and anxiety on the BDI [BPD+SA vs. HC: *p* < .001, *d* = 3.28; BPD-SA vs. HC: *p* < .001, *d* = 6.46], STAI-T [BPD+SA vs. HC: *p* < .001, *d* = 3.58; BPD-SA vs. HC: *p* < .001, *d* = 5.83] and STAXI-T [BPD+SA vs. HC: *p* < .001, *d* = 2.54; BPD-SA vs. HC: *p* < .001, *d* = 4.16] than HC participants.

**Table S3**

*Differences in demographical and psychopathological measures between HC and BPD participants with or without suicidal behavior*

|  | | HC (*n* = 20) | |  | BPD-SA (*n* = 8) | |  | BPD+SA (*n* = 13) | |  | Test statistic | |
| --- | --- | --- | --- | --- | --- | --- | --- | --- | --- | --- | --- | --- |
|  | | *n* | *%* |  | *n* | *%* |  | *n* | *%* |  | χ^2^(4*, N* = 41) | *p* |
| Education | |  |  |  |  |  |  |  |  |  | 3.26 | .515 |
|  | Basic | 0 | 0.00 |  | 0 | 0.00 |  | 1 | 0.08 |  |  |  |
|  | Intermediate | 5 | 0.25 |  | 3 | 0.33 |  | 5 | 0.38 |  |  |  |
|  | Advanced | 15 | 0.75 |  | 5 | 0.56 |  | 7 | 0.54 |  |  |  |
|  | | *M* | *SD* |  | *M* | *SD* |  | *M* | *SD* |  | *F*(2,38) | *p* |
| Age | | 26.81 | 4.89 |  | 24.95 | 3.38 |  | 26.98 | 7.35 |  | 0.39 | .68 |
| Intelligence (MWT-B-IQ) | | 108.20 | 9.20 |  | 109.00 | 14.01 |  | 108.62 | 15.52 |  | 0.01 | .99 |
| Borderline Personality (BSL-23) | | 1.55 | 2.09 |  | 41.88 | 22.93 |  | 40.08 | 19.64 |  | 35.70*** | <.001 |
| Attention deficit / Hyperactivity (ADHD-SR) | | 1.95 | 1.70 |  | 27.75 | 10.90 |  | 18.85 | 6.48 |  | 63.00*** | <.001 |
| Impulsivity (BIS-11) | | 52.85 | 7.69 |  | 74.00 | 14.18 |  | 74.69 | 11.24 |  | 22.41*** | <.001 |
| Anger (STAXI-T) | | 13.50 | 2.48 |  | 27.75 | 5.99 |  | 25.62 | 8.40 |  | 27.12*** | <.001 |
| Anxiety (STAI-T) | | 29.25 | 4.81 |  | 64.38 | 9.36 |  | 59.62 | 14.32 |  | 57.95*** | <.001 |
| Depression (BDI) | | 2.20 | 2.93 |  | 35.50 | 11.20 |  | 27.85 | 15.58 |  | 41.54*** | <.001 |
| *Note.* HC = healthy control participants; BPD-SA = borderline personality disorder participants without suicidal behavior; BPD+SA = borderline personality disorder participants with suicidal behavior; MWT-IQ = Multiple choice vocabulary test – Intelligence quotient ; BSL-23 = Borderline Symptom List 23 ; ADHD-SR = Attention Deficit Hyperactivity Disorder – Self Report Scale ; BIS-11 = Barratt Impulsiveness Scale Version 11 ; STAXI-T = State Trait Anger Expression Inventory – Trait Version ; STAI-T = State Trait Anxiety Inventory – Trait Version ; BDI = Beck Depression Inventory . *** *p* <.001. | | | | | | | | | | | | |

**References**

Barnow, S., Bernheim, D., Lischke, A., & Freyberger, H. J. (2014). *Fragebogen zur Selbstverletzung, Suizidalität und Medikamenten (SVUM)*. Department of Psychiatry and Psychotherapy. Greifswald.

Bohus, M., Kleindienst, N., Limberger, M. F., Stieglitz, R. D., Domsalla, M., Chapman, A. L., . . . Wolf, M. (2009). The short version of the Borderline Symptom List (BSL-23): development and initial data on psychometric properties. *Psychopathology, 42*(1), 32-39.

Hautzinger, M., Bailer, M., Worall, H., & Keller, F. (1995). *Beck-Depressions-Inventar (BDI). Testhandbuch*. Bern: Huber.

Laux, L., Glanzmann, P., Schaffner, P., & Spielberger, C. D. (1981). *Das State-Trait-Angstinventar*. Weinheim: Beltz.

Lehrl, S., Triebig, G., & Fischer, B. (1995). Multiple choice vocabulary test MWT as a valid and short test to estimate premorbid intelligence. *Acta Neurol Scand, 91*(5), 335-345.

Preuss, U. W., Rujescu, D., Giegling, I., Watzke, S., Koller, G., Zetzsche, T., . . . Moller, H. J. (2008). [Psychometric evaluation of the German version of the Barratt Impulsiveness Scale]. *Nervenarzt, 79*(3), 305-319.

Rosler, M., Retz, W., Retz-Junginger, P., Thome, J., Supprian, T., Nissen, T., . . . Trott, G. E. (2004). [Tools for the diagnosis of attention-deficit/hyperactivity disorder in adults. Self-rating behaviour questionnaire and diagnostic checklist]. *Nervenarzt, 75*(9), 888-895.

Schwenkmezger, P., Hodapp, V., & Spielberger, C. D. (1992). *Das State-Trait-Ärgerausdrucks-Inventar*. Bern: Huber.
